# Intermedin Inhibits the Ox-LDL–Induced Inflammation in RAW264.7 Cells by Affecting Fatty Acid–Binding Protein 4 Through the PKA Pathway

**DOI:** 10.3389/fphar.2021.724777

**Published:** 2021-12-01

**Authors:** Kai Liu, Rufeng Shi, Si Wang, Qi Liu, Hengyu Zhang, Xiaoping Chen

**Affiliations:** ^1^ Cardiology Department, West China Hospital, Sichuan University, Chengdu, China; ^2^ State Key Laboratory of Biotherapy, The Molecular Medicine Research Center, West China Hospital, Sichuan University, Chengdu, China

**Keywords:** intermedin (17-47)/adrenomedullin 2 (17-47), fatty acid–binding protein 4, inflammation, RAW264.7 cells, PKA

## Abstract

**Objectives:** Macrophages stimulated by oxidized low-density lipoprotein (ox-LDL) play an important role in the occurrence and progression of atherosclerosis. Fatty acid–binding protein 4 (FABP4), mainly existing in macrophages and adipocytes, can influence lipid metabolism and inflammation regulated by macrophages. Herein, we first established the connection between intermedin (IMD: a new peptide that has versatile biological activities in the cardiovascular system) and FABP4 and then investigated the influence of IMD on ox-LDL-induced changes in RAW264.7 macrophages line.

**Methods:** The bioinformatics analysis, such as gene ontology enrichment and protein–protein interactions, was performed. For ox-LDL–stimulated assays, RAW264.7 was first pretreated with IMD and then exposed to ox-LDL. To explore the cell signaling pathways of IMD on inflammatory inhibition, main signaling molecules were tested and then cells were co-incubated with relevant inhibitors, and then exposed/not exposed to IMD. Finally, cells were treated with ox-LDL. The protein and gene expression of FABP4, IL-6, and TNF-α were quantified by WB/ELISA and RT-qPCR.

**Results:** In the ox-LDL-stimulated assays, exposure of the RAW264.7 macrophages line to ox-LDL reduced cell viability and increased the expression of FABP4, as well as induced the release of IL-6 and TNF-α (all *p* < 0.05). On the other hand, IMD prevented ox-LDL–induced cell toxicity, FABP4 expression, and the inflammatory level in RAW264.7 (all *p* < 0.05) in a dose-dependent manner. The inhibition of FABP4 and the anti-inflammatory effect of IMD were partially suppressed by the protein kinase A (PKA) inhibitor H-89.

**Conclusion:** IMD can prevent ox-LDL–induced macrophage inflammation by inhibiting FABP4, whose signaling might partially occur via the PKA pathway.

## Introduction

Cardiovascular diseases (CVDs) are the leading causes of mortality worldwide, while atherosclerosis (AS) is a major risk factor for CVDs (Taggart, 2010). The World Health Organization has predicted that, by 2030, the total number of deaths from CVDs will increase by 27% (Roger, 2015). Previous studies proved that statin, a cholesterol-lowering drug, could effectively reduce AS development and CVDs outcomes; however, the residual risk remains (Ridker et al., 2010). Besides lipid metabolism, inflammation also has an important role in AS pathogenesis (Yudkin et al., 2000). Thus, exploring an effective anti-inflammatory strategy to reduce inflammation could attenuate AS and CVD prognosis.

Fatty acid–binding protein 4 (FABP4), a member of the fatty acid–binding protein (FABP) family, is a soluble carrier protein with low molecular weight and high affinity with fatty acids that mainly exists in macrophages and adipocytes (Furuhashi et al., 2014). Macrophages uptake oxidizes low-density lipoprotein (ox-LDL) cholesterol by scavenger receptors to form foam cells, which is a main pathological feature of AS (Seidelmann et al., 2014). FABP4 has an important role in both the transport and metabolism of fatty acids and inflammation mediated by macrophages, thus making it a promising target for AS treatment (Furuhashi et al., 2014).

Intermedin (IMD), also known as adrenomedullin 2, is a biologically active peptide that belongs to the calcitonin gene–related peptide (CGRP) superfamily (Roh et al., 2004). Combining different receptors and G protein, IMD has versatile biological activities in organisms, especially in the cardiovascular system (Zhang et al., 2018). Yet, whether IMD has a protective effect on macrophage-regulated inflammation and its mechanism remain unknown. Consequently, the present study aimed to test IMD as an anti-inflammatory treatment in the course of macrophages stimulated by ox-LDL and investigate the potential mechanisms.

## Materials and Methods

### Data Collection and Processing

All data sources that were included in the gene ontology (GO) enrichment analysis and protein–protein interaction (PPI) are publically available, as described below. Google Scholar and PubMed were used as search engines for protein identification. These proteins were proved or predicted to be the functional partners to the target protein. We downloaded the human gene annotations file from the GO website (http://geneontology.org) starting from May 01, 2021. The analysis was performed using STRING 11.0 (http://string-db.org/), g:Profiler (https://biit.cs.ut.ee/gprofiler/gost), and REVIGO (http://revigo.irb.hr/). The names of related proteins were searched in STRING, and codes were extracted. The codes used in STRING for PPI network and in g:Profiler for the GO analysis were molecular functions, cellular components, and biological processes. REVIGO was used for visualized illustration of predicted interactions between target proteins.

### Materials

RAW264.7 cells were purchased from American Type Culture Collection. Dulbecco's modified Eagle's medium (DMEM), fetal bovine serum (FBS), and phosphate-buffered saline were obtained from Invitrogen (Carlsbad, CA, United States). Dimethyl sulfoxide (DMSO) and Cell Counting Kit-8 (CCK-8) were from KeyGEN BioTECH (Nanjing, China). IMD was acquired from Phoenix BioTECH (Beijing, China). Ox-LDL was obtained from Peking Union Biology (Beijing, China), and superoxide dismutase (SOD) and malondialdehyde (MDA) assay kits were purchased from Jiancheng Bioengineering Institute (Nanjing, China). Bovine serum albumin was purchased from Sigma-Aldrich (Saint Louis, MO, United States). The protein kinase A inhibitor (PKAI; H-89), protein kinase G inhibitor (PKGI; KT5823), and p38-mitogen–activated protein kinase inhibitor (p38-MAPKI; SB203580) were obtained from Beyotime Biotechnology (Shanghai, China). A PrimeScript™ RT reagent kit with gDNA Eraser and SYBR Premix EX Taq™ II were purchased from TaKaRa Bio (Tokyo, Japan). TNF-α and IL-6 kits for enzyme-linked immunosorbent assay (ELISA) were purchased from Invitrogen, as was 2′,7′-dichlorodihydrofluorescein diacetate. The first antibody for FABP4 (ab92501) and β-actin (ab8226) was bought from Abcam.

### Cell Culture and Treatments

Cells were maintained in DMEM (high glucose) supplemented with 10% FBS, 100 U/ml penicillin, and 100 μg/ml streptomycin in 95% air and 5% CO_2_ at 37°C. When cells presented monolayer and confluence up to 70%, they were pretreated with various concentrations (0, 20, 40, and 80 nmol/L) of IMD for 24 h and were subsequently incubated with 50 μg/ml ox-LDL for 24 h. To explore the cell signaling pathways of IMD for inhibition of inflammation and oxidative stress, sub-confluence cells were co-incubated/not co-incubated with H-89 (1 μmol/L, PKAI), KT5823 (1 μmol/L, PKGI), and SB203580 (1 μmol/L, p38-MAPKI) for 30 min, and then exposed/not exposed to 80 nmol/L IMD for 24 h. Finally, cells were treated with 50 μg/ml ox-LDL for another 24 h. The cells treated with equal amounts of DMSO were used as controls.

### Cell Viability

RAW264.7 cells were plated at a density of 1 × 10^4^ per well in a 96-well plate and incubated at 37°C. The cells were treated with different concentrations of IMD (0, 20, 40, and 80 nmol/L) for 24 h, after which the CCK-8 reagent (Nanjing, China) was added for 4 h. Thereafter, the absorbance at 450 nm was measured using an ELISA microplate reader (Benchmark; BioRad Laboratories, CA, United States). Cell viability was calculated relative to that of the control group. All experiments were performed in triplicate.

### SOD Measurement

The cells were scraped and operated according to the SOD detection kit. The cells were centrifuged at 1,500 rpm/min for 10 min, and then precipitated. After the buffer was added, the cells were sonicated. The reagent was added to the cells according to the kit instruction and incubated at 37°C for 20 min. The absorption was read at 450 nm.

### MDA Measurement

The pretreatment of the cells is the same as mentioned in SOD measurement, followed by heating at 95°C for 20 min. The reagents required for the experiment were prepared and tested as the MDA kit instructed. The absorbance was read at 530 nm.

### Western Blot

The method was previously described by Liao et al. (2017). In short, cells were lysed by using a 4× Sodium dodecyl sulfate (SDS) sample buffer and then the samples were boiled. 20 μg of the extract was loaded onto 12% SDS–polyacrylamide gel (PAGE) and transferred to polyvinylidene fluoride membranes (Bio-Rad, United States). Immunoblotting was performed using the first antibody overnight at 4°C. β-actin was used as the control protein. The blots were detected using an enhanced chemiluminescence reagent (Thermo, United States). The amount of each target was normalized by the level of β-actin in each sample. The experiments were repeated at least three times.

### Enzyme-Linked Immunosorbent Assay

Cell supernatants (100 μl) were used to quantify IL-6 and TNF-α using TaKaRa Bio ELISA kits (TaKaRa, Japan) according to manufacturer's instructions. Briefly, human TNF-α and IL-6 antibodies were coated on 96-well plates followed by incubation for 2 h at room temperature with cell supernatants. Then, 100 µl of biotin-conjugated human TNF-α or IL-6 was added to the solution in each well, followed by incubation for 1 h at room temperature. Then, 100 µl of 1× streptavidin–horseradish peroxidase solution was placed in each well and incubated for 30 min at room temperature. A stabilized chromogen was added to each well, followed by incubation for 30 min in the dark at room temperature. Stop Solution (100 µl) was added to each well. The side of the plate was tapped to allow mixing. Finally, the plate was read, and a standard curve was generated.

### Quantitative Real-time polymerase chain reaction (RT-PCR)

Total RNA was extracted from cultured RAW264.7 cells using MiniBEST Universal RNA Extraction Kit (TaKaRa, Japan), followed by reverse transcription into cDNA using the PrimeScript™ preamplification system (TaKaRa, Japan) according to the manufacture's protocol. PCR was then performed to estimate the expression of FABP4, IL-6, and TNF-α. The sequence-specific primers are shown in Table 1. After an initial denaturation for 5 min at 94°C, followed by 40 cycles of denaturation (95°C for 1 min), annealing (60°C for 1 min), extension (72°C for 1 min) ensued with a final extension of 72°C for 10 min. The β-actin mRNA amplified from the same samples served as the control.

**TABLE 1:** The sequence-specific primers for RT-PCR.

Gene	Forward primer 5′ to 3′	Reverse primer 5′ to 3′
β-actin	CTA​AGG​CCA​ACC​GTG​AAA​G	ACC​AGA​GGC​ATA​CAG​GGA​CA
FABP4	TCA​CCT​GGA​AGA​CAG​CTC​CT	AAT​CCC​CAT​TTA​CGC​TGA​TG
IL-6	AAC​GAT​GAT​GCA​CTT​GCA​GA	GAG​CAT​TGG​AAA​TTG​GGG​TA
TNF-α	ACA​CTC​AGA​TCA​TCT​TCT​CAA​AAT​TCG	GTG​TGG​GTG​AGG​AGC​ACG​TAG​T

### RNA Interference

Lipofectamine RNAiMAX (Invitrogen, United States) targeting FABP4 and a scramble controlled small interfering RNA (scramble siRNA) were used to transfect RAW264.7 cells (2.5 × 10^5^ per well, 6-well plates) transiently for 4 h. The primer sequences for FABP4 siRNA were sense, 5′-AUA​CUG​AGA​UUU​CCU​UCA​U-3′, and antisense, 5′-GGU​GGA​AUG​CGU​CAU​GAA​A-3′.

### Statistical Analyses

All experiments were performed at least three times, and the results are expressed as mean ± standard deviation of the mean (SD). One-way analysis of variance was used to analyze the group differences. *p* < 0.05 was considered as a statistically significant difference.

## Results

### IMD Interacts With FABP4 and Regulates the Inflammatory Response

Data mining suggested the ten most related proteins to IMD, i.e., ADM, CALCRL, RAMP1, RAMP2, RAMP3, CALCA, IAPP, CALCR, CALCB, and POMC (Supplementary Figure S1A). Most of them belonged to the CGRP superfamily, which binds its ligand and activates multiple biological processes (Supplementary Figures S1B,C). Then, FABP4 was added into the analysis, revealing the interactions between IMD and FABP4 (Supplementary Figures S2A,B). Some of their biological processes were associated with lipid metabolism and inflammatory response (Supplementary Figure S2C).

### IMD Ameliorates Ox-LDL–Induced Cytotoxic Effect in RAW264.7

**FIGURE 1:**
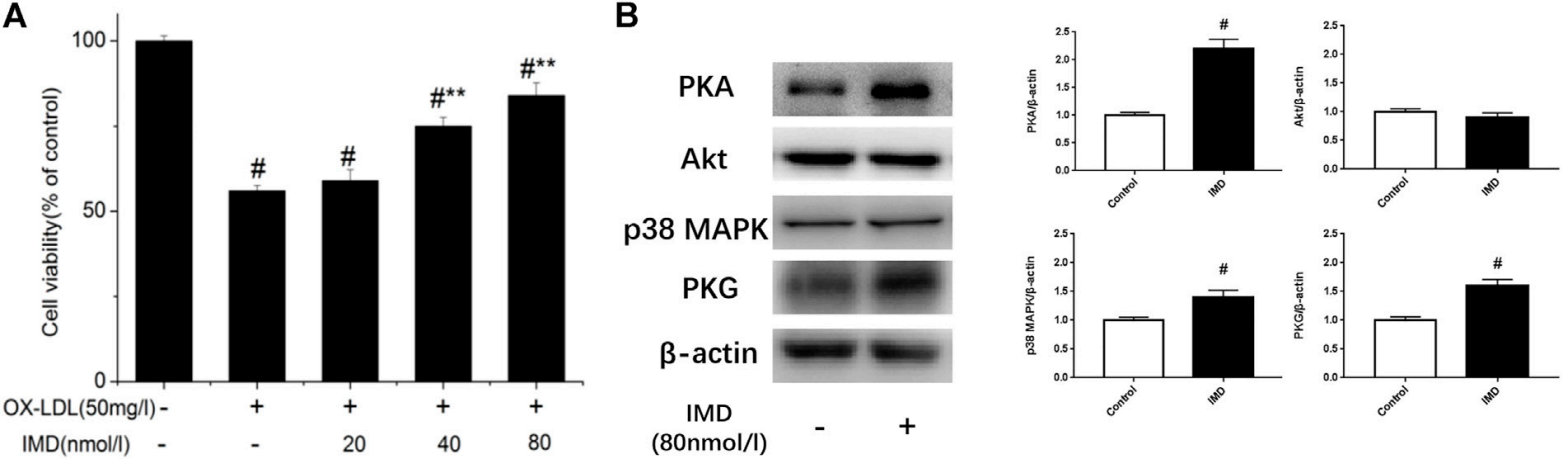
Dose-dependent protective effect of IMD on ox-LDL–induced macrophages and its signaling pathway. ^#^ Compared with the control group (*p* < 0.05); ** compared to the ox-LDL alone group (*p* < 0.05).

**FIGURE 2:**
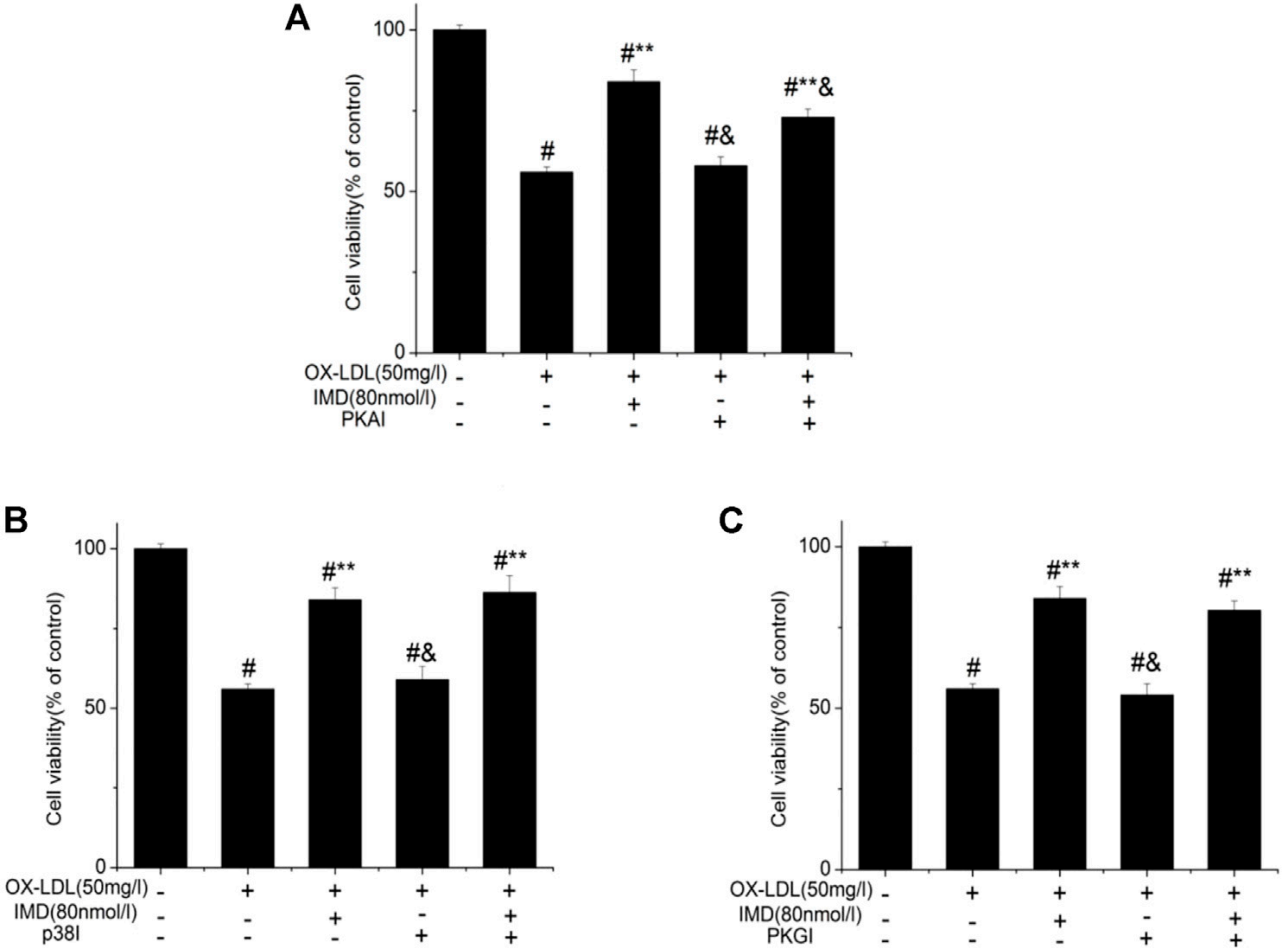
Protective effect of IMD partially through the PKA pathway. ^#^ Compared with the control group (*p* < 0.05); ** compared to the ox-LDL alone group (*p* < 0.05); ^&^ compared to the ox-LDL + IMD group (*p* < 0.05).

Compared with the blank control group, the cell viability of 50 mg/L ox-LDL intervention treatment was significantly reduced (Figure 1A). However, when cells were pretreated with IMD, the cell viability gradually recovered in a dose-dependent manner compared to the ox-LDL alone intervention group (Figure 1A), which was in accordance with the oxidation activity (Table 2). Thus, it indicates that IMD could counteract the damage to macrophages that was stimulated by ox-LDL. Among IMD's main signaling molecules, Western blot showed that PKA, p38-mitogen–activated protein kinase, and protein kinase G were altered after IMD intervention (Figure 1B). Moreover, when 10 μmol/L H-89 (PKAI) was pretreated with 80 nmol/L IMD, the cytoprotective effect was partially inhibited (Figure 2A). Neither the 1 μmol/L KT5823 (p38 inhibitor) group nor the 1 μmol/L SB203580 (PKGI) group showed this phenomenon (Figures 2B,C).

**TABLE 2:** Cell MDA and SOD activity.

Activity	Control group	Ox-LDL group	Ox-LDL + IMD group
SOD (μ/ml)	14.51 ± 1.13	12.47 ± 0.97^a^	13.97 ± 1.07^b^
MDA (nmol/ml)	2.89 ± 0.05	3.73 ± 0.08^a^	3.06 ± 0.09^b^

Control group was added carrier medium, ox-LDL group 50 mg/L ox-LDL; ox-LDL + IMD group 50 mg/L ox-LDL and 40 nmol/L IMD. Values are presented as the mean ± standard error of the mean.

a *p* < 0.01 vs. control group.

b *p* < 0.01 vs. ox-LDL group.

### IMD Inhibits Upregulated FABP4 in RAW264.7 Stimulated by Ox-LDL

**FIGURE 3:**
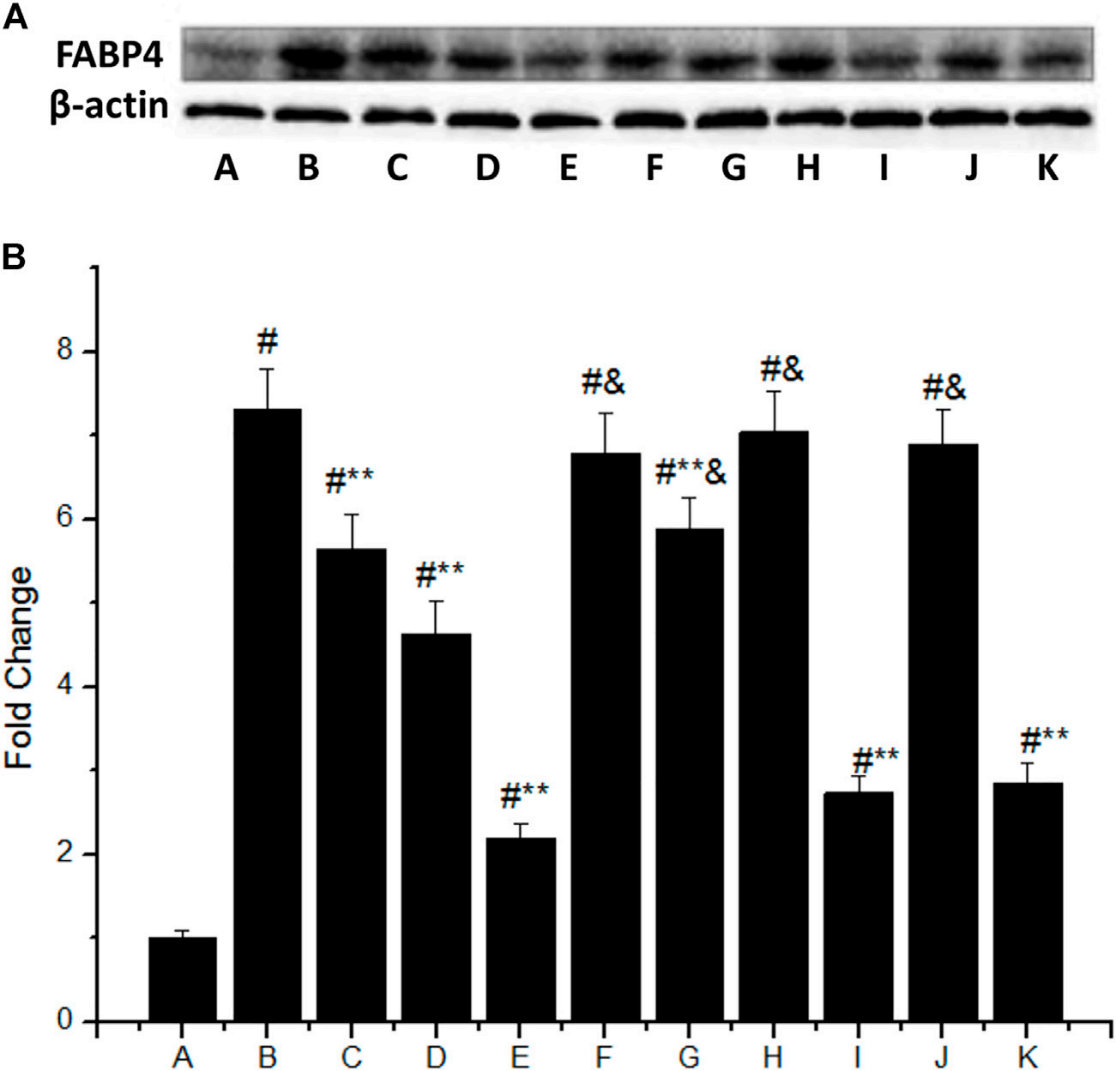
IMD inhibits FABP4 protein expression dose-dependently in ox-LDL–stimulated macrophages partially through the PKA pathway. **(A)** Western blot for FABP4 protein in RAW264.7; **(B)** Quantitative band density analysis according to the gray image scanning, and normalization of all bands to housekeeping protein β-actin. **(A)** control group; **(B)** ox-LDL group; **(C)** ox-LDL+20 nm IMD group; **(D)** ox-LDL+40 nm IMD group; **(E)** ox-LDL+80 nm IMD group; **(F)** ox-LDL + PKAI group; **(G)** ox-LDL + PKAI+80 nm IMD group; **(H)** ox-LDL + p38 inhibitor group; **(I)** ox-LDL + p38 inhibitor+80 nm IMD group; **(J)** ox-LDL + PKGI group; **(K)** ox-LDL + PKGI +80 nm IMD group. ^#^ Compared with group A (*p* < 0.05); ** compared to group B (*p* < 0.05); ^&^ compared to group E (*p* < 0.05).

The FABP4 expression was significantly increased in macrophages stimulated with 50 mg/L ox-LDL (Figure 3A), which was 7.3 times higher than that of the control group (group B vs. group A, *p* < 0.05). However, IMD could downregulate the mentioned incremental FABP4 in a concentration-dependent manner, where the FABP4 expression from low to high IMD concentration was 5.65, 4.63, and 2.2 times compared to the control group (compared with group B, all *p* < 0.05). The 80 nm IMD + PKAI group was 5.89 times compared to the control group (group G vs. group A, *p* < 0.05), while the 80 nm IMD alone group was 2.2 times compared to the control group (group E vs. group A, *p* < 0.05), thus indicating that the PKAI could partially inhibit this effect of IMD, while PKGI and p38 inhibitor did not block such inhibitory effect (Figure 3B).

**FIGURE 4:**
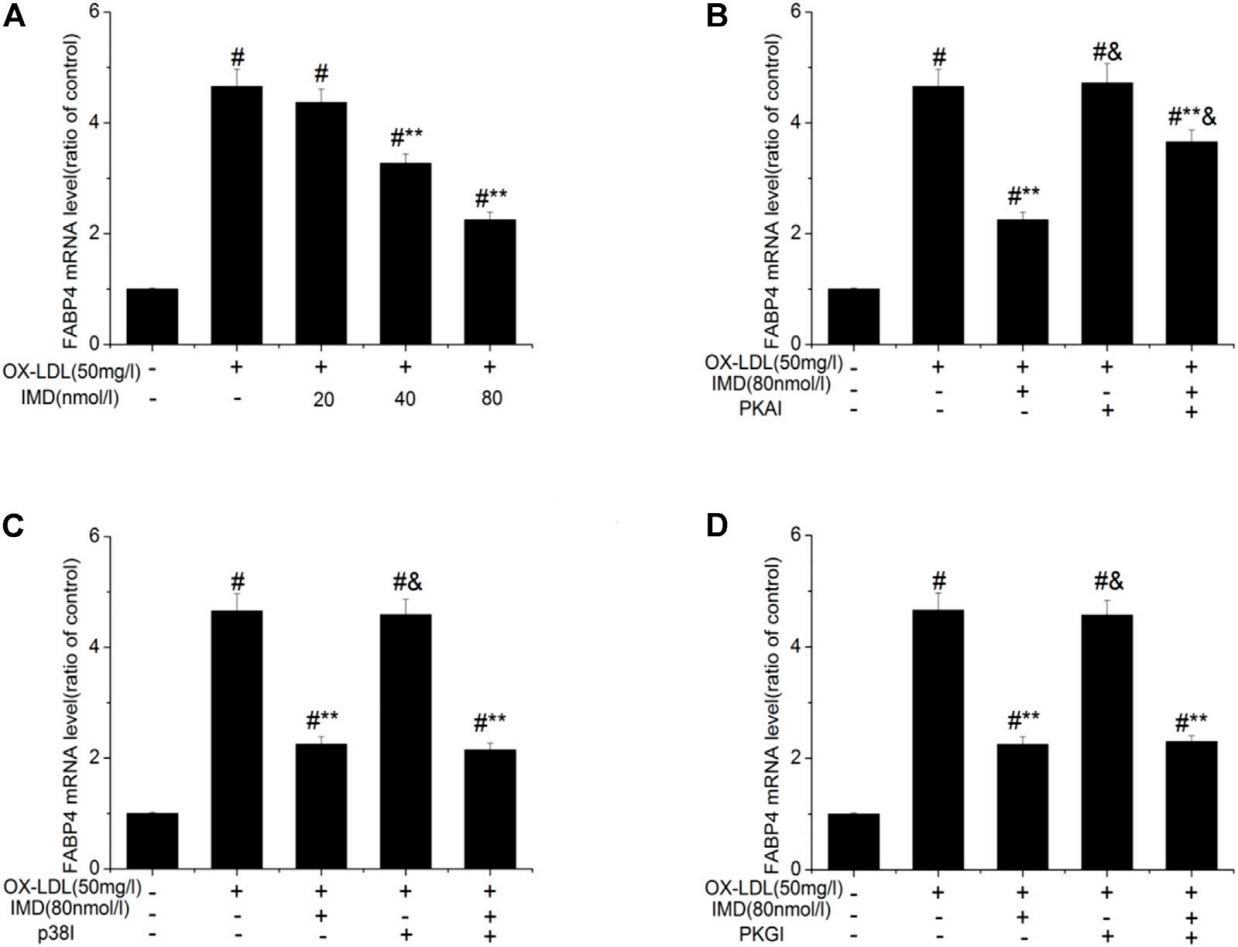
IMD inhibits the mRNA expression of FABP4 in ox-LDL–induced macrophages. ^#^ Compared with the control group (*p* < 0.05); ** compared to the ox-LDL alone group (*p* < 0.05); ^&^ compared to the ox-LDL +IMD group (*p* < 0.05).

Accordingly, RT-PCR revealed that the mRNA of FABP4 in the ox-LDL group was significantly higher than in the control group (*p* < 0.05). The mRNA of FABP4 in the 40 nmol/L IMD group was lower than in the ox-LDL group (3.27 ± 0.17 vs. 4.66 ± 0.31, *p* < 0.05), while the 80 nmol/L IMD group was lower than in the ox-LDL group (2.25 ± 0.14 vs. 4.66 ± 0.31, *p* < 0.05), which indicated that IMD could reduce the expression of the FABP4 gene (Figure 4A).

In addition, the mRNA of FABP4 in the PKAI group was increased compared with the ox-LDL + IMD group (Figure 4B; 3.66 ± 0.21 vs. 2.25 ± 0.14, *p* < 0.05), thus indicating that PKAI could partially inhibit IMD downregulation of the FABP4 gene expression. The p38 and PKGI groups had no such changes (Figures 4C,D).

### IMD Reduced Ox-LDL–Stimulated Production of IL-6 and TNF-α

**FIGURE 5:**
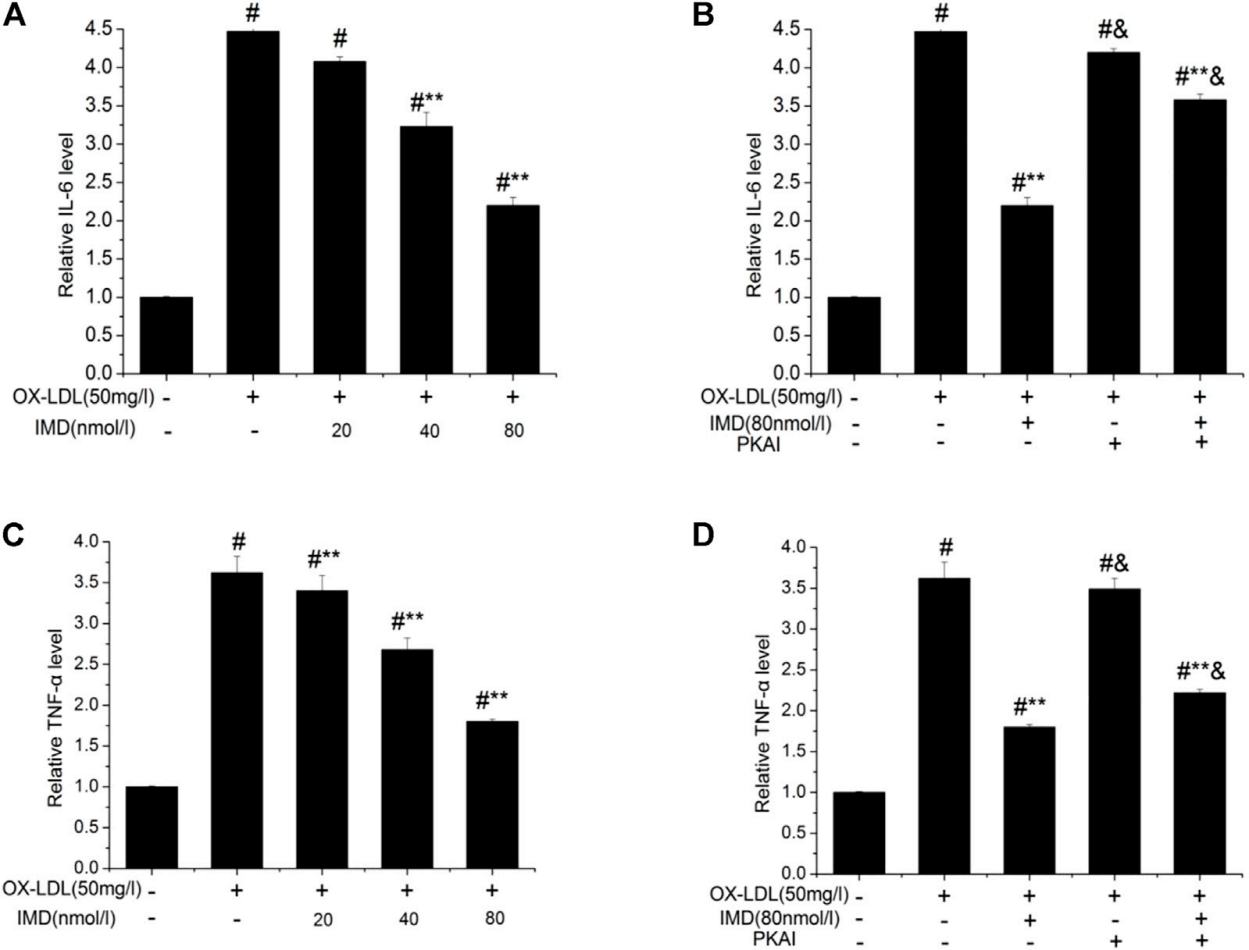
IMD inhibits the protein expression of IL-6 and TNF-α in ox-LDL–induced macrophages (ELISA). ^#^ Compared with the control group (*p* < 0.05); ** compared to the ox-LDL alone group (*p* < 0.05); ^&^ compared to the ox-LDL +IMD group (*p* < 0.05).

**FIGURE 6:**
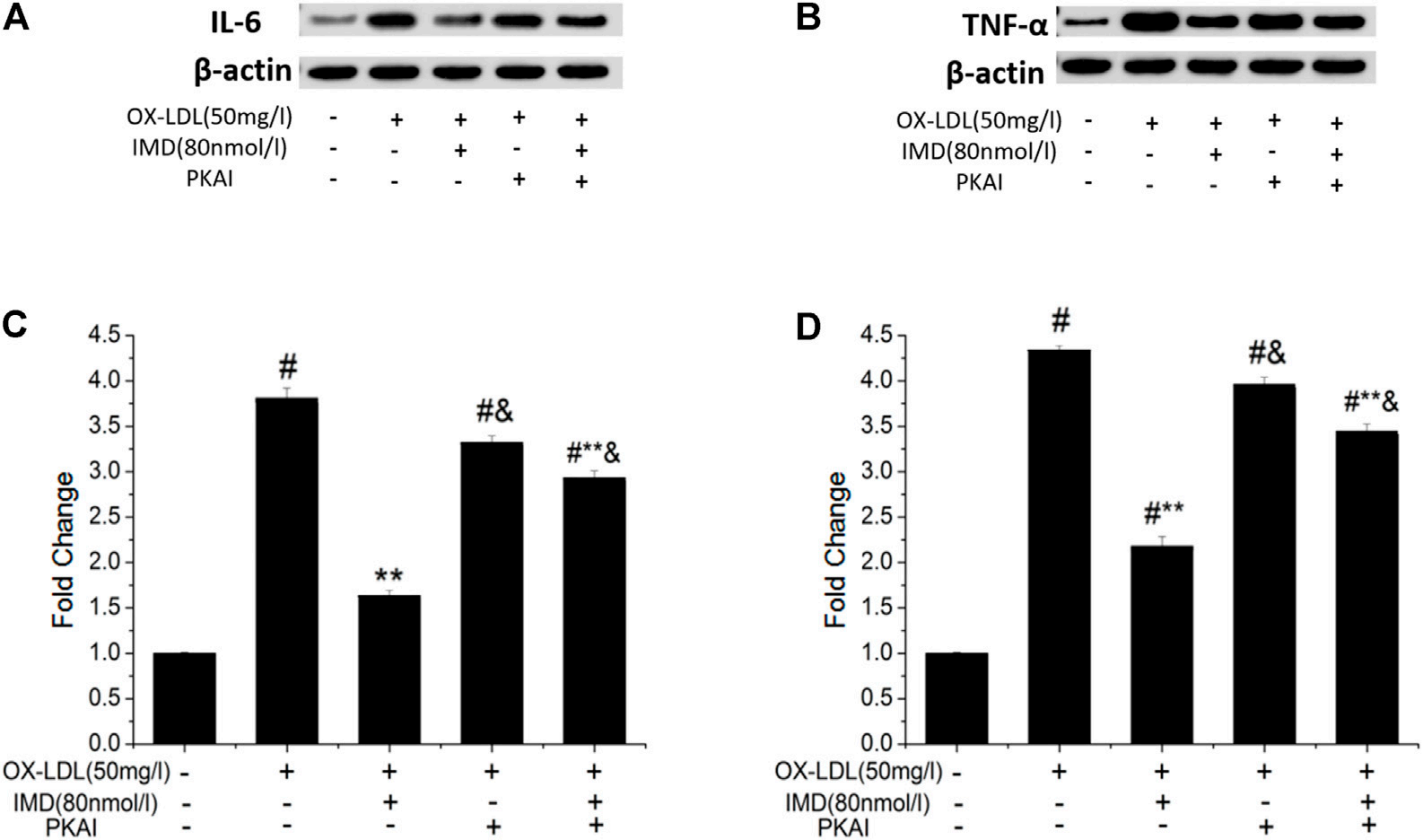
IMD inhibits the protein expression of IL-6 and TNF-α in ox-LDL–induced macrophages (WB). ^#^ Compared with the control group (*p* < 0.05); ** compared to the ox-LDL alone group (*p* < 0.05); ^&^ compared to the ox-LDL +IMD group (*p* < 0.05).

**FIGURE 7:**
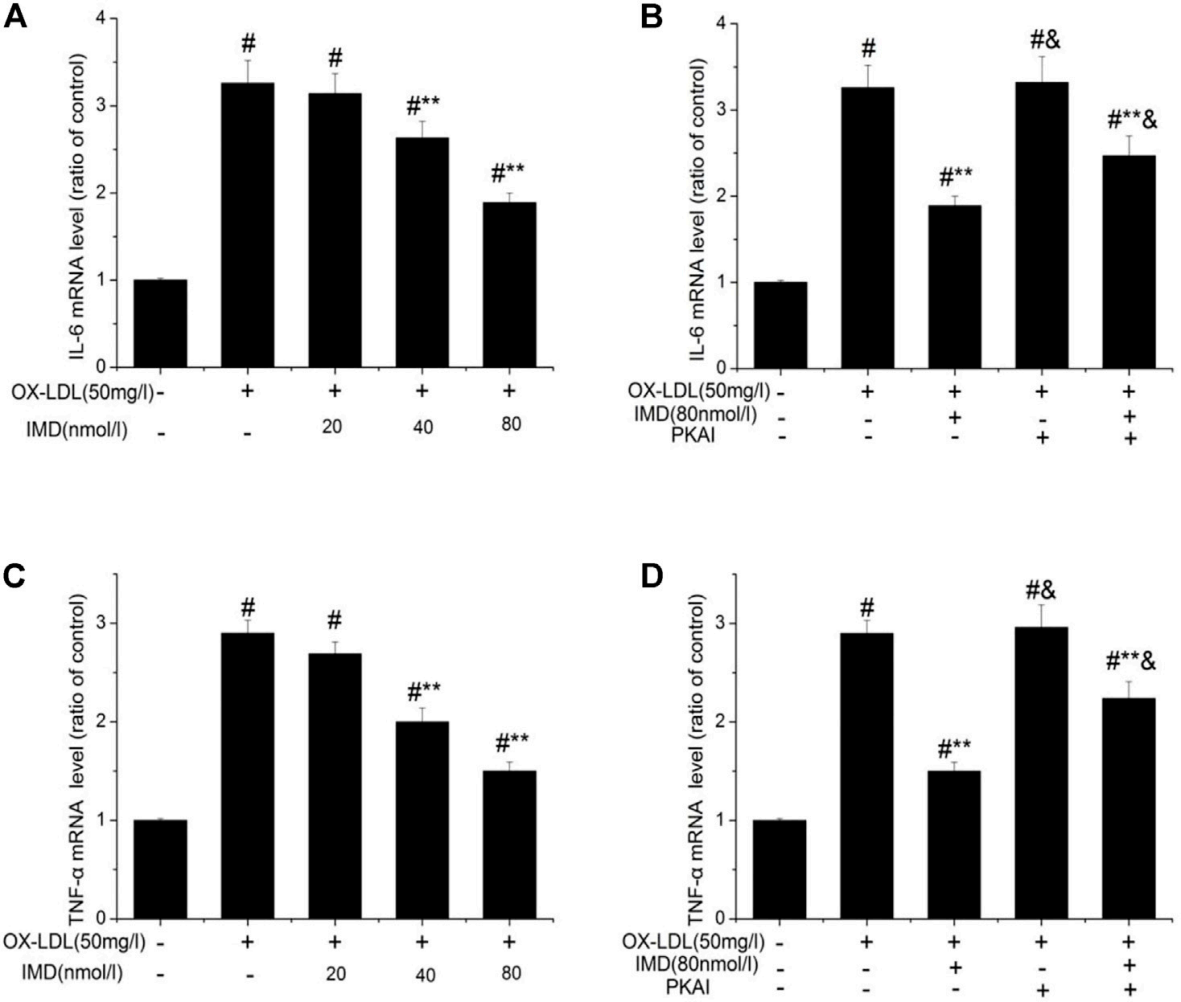
IMD inhibits the mRNA expression of IL-6 and TNF-α in ox-LDL–induced macrophages. ^#^ Compared with the control group (*p* < 0.05); ** compared to the ox-LDL alone group (*p* < 0.05); ^&^ compared to the ox-LDL +IMD group (*p* < 0.05).

The anti-inflammatory effects of IMD on ox-LDL–induced production of IL-6 or TNF-α in the RAW264.7 cells were evaluated by ELISA, Western blot (WB), and RT-PCR. The protein and mRNA expression of both IL-6 and TNF-α were obviously increased in the cells treated with ox-LDL alone compared to the unstimulated cells (Figures 6, 7, all *p* < 0.05). By contrast, IMD treatment reduced ox-LDL–stimulated production of IL-6 and TNF-α in a dose-dependent manner (Figures 5, 6, all *p* < 0.05). Considering the protein level, the 40 nmol/L IMD group had significantly decreased IL-6 (Figure 5A; 3.23 ± 0.27 vs. 4.47 ± 0.11, by ELISA, *p* < 0.05) and TNF-α (Figure 5C; 2.71 ± 0.28 vs. 3.62 ± 0.34, by ELISA, *p* < 0.05) compared with the ox-LDL group, while 80 nmol/L showed a similar but more dramatic effect (Figures 5A,C; IL-6: 2.21 ± 0.21 vs. 4.47 ± 0.11; TNF-α:1.76 ± 0.09 vs. 3.62 ± 0.34, by ELISA, both *p* < 0.05). At the mRNA level, the 40 nmol/L and 80 nmol/L IMD groups had significantly decreased IL-6 (Figure 7A; 1.89 ± 0.11 vs. 2.63 ± 0.19 vs. 3.26 ± 0.26, *p* < 0.05) and TNF-α (Figure 7C; 1.5 ± 0.1 vs. 2.0 ± 0.12 vs. 2.9 ± 0.13, *p* < 0.05), respectively. Similarly, the anti-inflammatory effect of IMD on the ox-LDL–induced release of IL-6 and TNF-α was inhibited when macrophages were pretreated with PKAI.

### IMD Inhibit Inflammation Through FABP4

**FIGURE 8:**
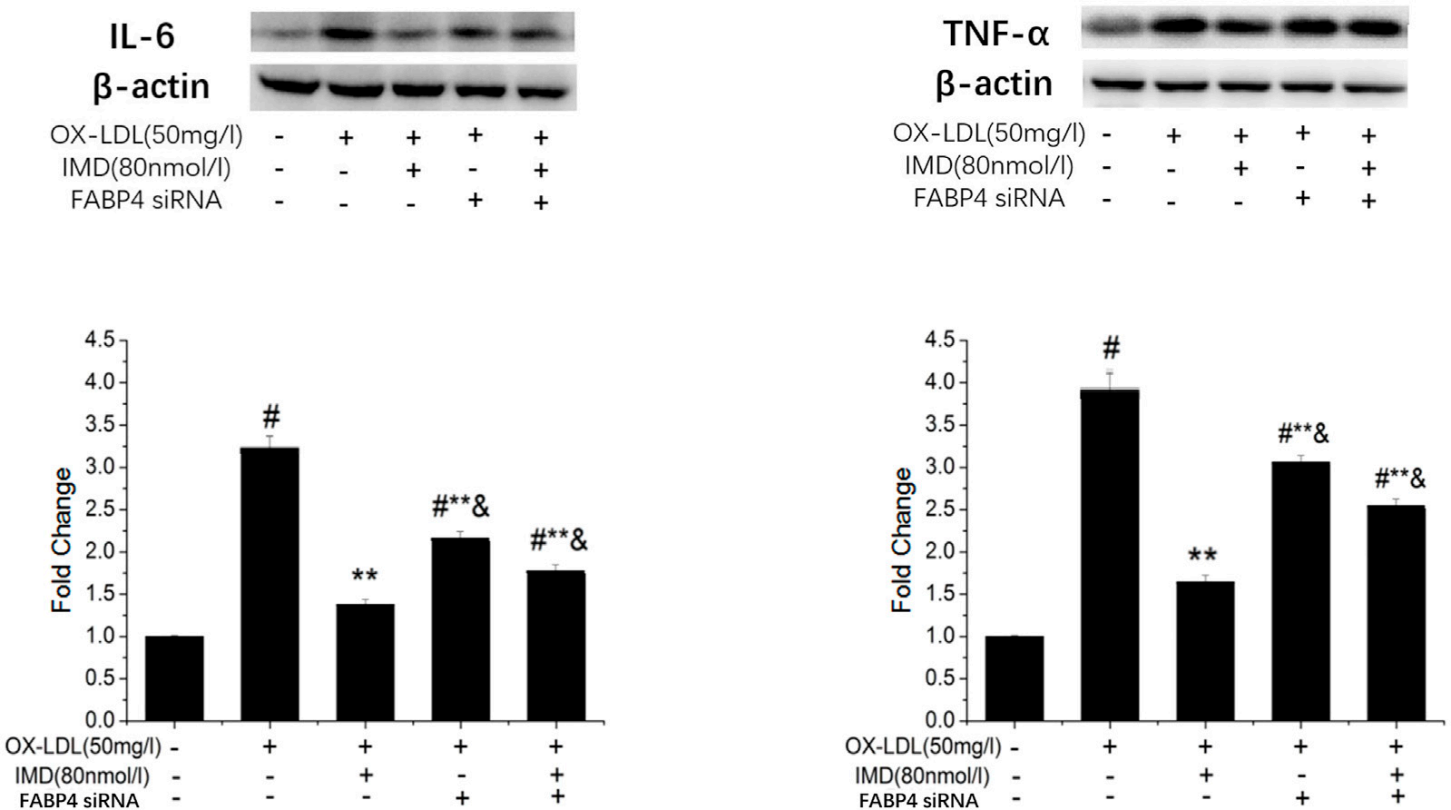
IMD inhibits the protein expression of IL-6 and TNF-α through the FABP4 pathway. ^#^ Compared with the control group (*p* < 0.05); ** compared to the ox-LDL alone group (*p* < 0.05); ^&^ compared to the ox-LDL +IMD group (*p* < 0.05).

The FABP4 inhibitor was used to validate whether IMD fulfilled its anti-inflammatory effect via the FABP4 pathway. We used siRNA to knockdown FABP4, and its mRNA expression was significantly decreased (Supplementary Figure S5). Compared with the IMD intervention group, the production of IL-6 and TNF-α were almost recovered to the ox-LDL simulated level when FABP4 siRNA was pretreated, thus suggesting that IMD partially inhibited these inflammatory factors via the FABP4 pathway (Figure 8).

## Discussion

Our study showed that IMD counteracted the ox-LDL–induced injury in RAW264.7 cells by inhibiting FABP4. However, this process was partially inhibited by the PKAI. This data suggested that IMD inhibits the ox-LDL–induced inflammation in RAW264.7 cells by affecting fatty acid–binding protein 4 through the PKA pathway.

AS, one of the main causes of CVDs and cerebrovascular diseases, is considered a chronic inflammatory disease (Bobryshev et al., 2016). Lipid metabolism disorders are the basis of AS, triggering the subsequent inflammatory response regulated by macrophages (Bobryshev et al., 2016). After vascular endothelial cells are damaged, the vascular endothelium secretes adhesion molecules to mediate the adhesion of monocytes and lymphocytes. Once monocytes adhere, they migrate into the subintimal space of the damaged endothelium under the action of chemokines and transform into macrophages. Macrophages engulf ox-LDL-C–based cholesterol through CD36 and scavenger receptor A, promoting monocyte-derived macrophages to transform to foam cells and secrete proinflammatory cytokines, along with atherosclerotic lesions progress. Therefore, macrophages and inflammation that they regulate may be used as a promising target for AS therapy.

IMD and its receptor complex comprise calcitonin receptor-like receptor (CRLR), while receptor activity–modifying protein (RAMP) 1, 2, and 3 are highly expressed in the vasculature (Zhang et al., 2018). Coupling different G proteins, IMD presents diverse biological effects such as anti-inflammatory, anti-oxidation, and anti-apoptosis via a different signaling pathway. Consequently, IMD has a regulatory effect on cardiovascular homeostasis. Our previous study showed that IMD ameliorates AS in ApoE null mice (Zhang et al., 2012). Besides, a study suggested that IMD could prevent the atherosclerotic lesions progression by inhibiting ERS-CHOP–mediated apoptosis and inflammasome in macrophages (Ren et al., 2021). However, it remains unclear whether other mechanisms are involved.

Previous studies have proved that FABP4 is an important factor in the development of AS due to metaflammation. FABP4 knockout can prevent the development of AS in ApoE^−/−^ mice (Makowski et al., 2001). On the other hand, the FABP4 expression is significantly upregulated during the process of ox-LDL–induced macrophages forming foam cells, which might be because ox-LDL induces FABP4 gene expression by activating the nuclear factor NF-κB and protein kinase C pathway (Fu et al., 2002). Other studies also suggested that peroxidase proliferator–activated receptor γ (PPARγ) might be involved (Fu et al., 2002; Makowski et al., 2005). Bone marrow transplantation studies have confirmed that this effect should be attributed to FABP4 in macrophages rather than to adipocytes for promoting AS lesion formation (Rolph et al., 2006). Our previous study showed that IMD enhanced the expression of ABCA1, a cholesterol efflux pathway–associated protein, and cholesterol efflux in macrophages (Liao et al., 2017), while other studies demonstrated that FABP4 gene deletion could upregulate ABCA1 (Chawla et al., 2001). Therefore, IMD increasing ABCA1 to regulate cholesterol efflux by inhibiting FABP4 expression might explain the alleviation of AS.

The mechanism of FABP4 resulting in AS is due to its direct effect on lipid metabolism and its ability to mediate inflammatory responses. FABP4 binds monounsaturated fatty acids and polyunsaturated fatty acids that prevent the activation of PPARγ. On the other hand, it also links Liver X receptor α, thus disrupting the expression of PPARγ-dependent genes. This reduces the expression of SIRT3 and UCP2, leading to an enhanced production of ROS and inflammatory responses (Korbecki and Bajdak-Rusinek, 2019). *In vitro* study demonstrated that FABP4 knockout significantly reduced the expression of IL-1β, IL-6, TNF-α, and MCP-1 (Fu et al., 2002; Ren et al., 2021). This is consistent with our results revealing that IMD reduced the generation of IL-6 and TNF-α in macrophages stimulated by ox-LDL, which might be another result ensuing the inhibition of FABP4. The mechanism might be based on FABP4 activating IκB kinase and NF-κB activity, which in turn upgrades the expression of cyclooxygenase 2 and inducible NO synthase and the production of inflammatory cytokines (Fu et al., 2002). Another pathway lies in FABP4 that has a positive feedback relationship with JNK and activator protein-1 (AP-1), which promote the inflammatory response induced by lipopolysaccharide (Hui et al., 2010).

A common receptor of the CGRP superfamily is the calcitonin receptor-like receptor/receptor activation–modified protein complex (CRLR/RAMP) (Zhang et al., 2018). CRLR belongs to the G protein–coupled receptor superfamily and has complex downstream signaling pathways, such as the AC-cAMP/PKA, L-arginine–NO–cGMP, and Gq/11-PLC (Kandilci et al., 2008; Dai et al., 2012; Li et al., 2013). Nevertheless, the pathway through which IMD inhibits FABP4 and the inflammatory response remains unknown. In our study, three signaling pathways were verified, and only PKAI significantly blocked the above effects, suggesting that IMD exerted these functions through the PKA signaling pathway, which is consistent with our previous findings (Rolph et al., 2006).

This study has a few limitations. First, our data must be confirmed to be *in vivo*. Second, whether IMD causes vascular inflammatory disease and whether other mechanisms are involved needs to be further addressed by future studies.

In conclusion, IMD could prevent ox-LDL–induced macrophages inflammation by inhibiting FABP4, where signaling might partially occur via the PKA pathway.

## Data Availability

The raw data supporting the conclusion of this article will be made available by the authors, without undue reservation.

## References

[B1] Bobryshev Y. V. Ivanova E. A. Chistiakov D. A. Nikiforov N. G. Orekhov A. N. (2016). Macrophages and Their Role in Atherosclerosis: Pathophysiology and Transcriptome Analysis. Biomed. Res. Int. 2016, 9582430. 10.1155/2016/9582430 27493969 PMC4967433

[B2] Chawla A. Boisvert W. A. Lee C. H. Laffitte B. A. Barak Y. Joseph S. B. (2001). A PPAR Gamma-LXR-ABCA1 Pathway in Macrophages Is Involved in Cholesterol Efflux and Atherogenesis. Mol. Cel 7, 161–171. 10.1016/s1097-2765(01)00164-2 11172721

[B3] Dai X. Y. Cai Y. Mao D. D. Qi Y. F. Tang C. Xu Q. (2012). Increased Stability of Phosphatase and Tensin Homolog by Intermedin Leading to Scavenger Receptor A Inhibition of Macrophages Reduces Atherosclerosis in Apolipoprotein E-Deficient Mice. J. Mol. Cel Cardiol 53, 509–520. 10.1016/j.yjmcc.2012.07.006 22841663

[B4] Fu Y. Luo N. Lopes-Virella M. F. Garvey W. T. (2002). The Adipocyte Lipid Binding Protein (ALBP/aP2) Gene Facilitates Foam Cell Formation in Human THP-1 Macrophages. Atherosclerosis 165, 259–269. 10.1016/s0021-9150(02)00305-2 12417276

[B5] Furuhashi M. Saitoh S. Shimamoto K. Miura T. (2014). Fatty Acid-Binding Protein 4 (FABP4): Pathophysiological Insights and Potent Clinical Biomarker of Metabolic and Cardiovascular Diseases. Clin. Med. Insights Cardiol. 8, 23–33. 10.4137/CMC.S17067 PMC4315049 25674026

[B6] Hui X. Li H. Zhou Z. Lam K. S. Xiao Y. Wu D. (2010). Adipocyte Fatty Acid-Binding Protein Modulates Inflammatory Responses in Macrophages through a Positive Feedback Loop Involving C-Jun NH2-terminal Kinases and Activator Protein-1. J. Biol. Chem. 285 (14), 10273–10280. 10.1074/jbc.M109.097907 20145251 PMC2856232

[B7] Kandilci H. B. Gumusel B. Lippton H. (2008). Intermedin/adrenomedullin-2 (IMD/AM2) Relaxes Rat Main Pulmonary Arterial Rings via cGMP-dependent Pathway: Role of Nitric Oxide and Large Conductance Calcium-Activated Potassium Channels (BK(Ca)). Peptides 29 (8), 1321–1328. 10.1016/j.peptides.2008.04.008 18538894

[B8] Korbecki J. Bajdak-Rusinek K. (2019). The Effect of Palmitic Acid on Inflammatory Response in Macrophages: an Overview of Molecular Mechanisms. Inflamm. Res. 68, 915–932. 10.1007/s00011-019-01273-5 31363792 PMC6813288

[B9] Li P. Sun H. J. Han Y. Wang J. J. Zhang F. Tang C. S. (2013). Intermedin Enhances Sympathetic Outflow via Receptor-Mediated cAMP/PKA Signaling Pathway in Nucleus Tractus Solitarii of Rats. Peptides 47, 1–6. 10.1016/j.peptides.2013.05.002 23816795

[B10] Liao H. Wan S. Zhang X. Shi D. Zhu X. Chen X. (2017). Intermedin Ameliorates Atherosclerosis by Increasing Cholesterol Efflux through the cAMP-PKA Pathway in Macrophage RAW264.7 Cell Line. Med. Sci. Monit. 23, 5462–5471. 10.12659/msm.907298 29146892 PMC5702107

[B11] Makowski L. Boord J. B. Maeda K. Babaev V. R. Uysal K. T. Morgan M. A. (2001). Lack of Macrophage Fatty-Acid-Binding Protein aP2 Protects Mice Deficient in Apolipoprotein E against Atherosclerosis. Nat. Med. 7 (6), 699–705. 10.1038/89076 11385507 PMC4027052

[B12] Makowski L. Brittingham K. C. Reynolds J. M. Suttles J. Hotamisligil G. S. (2005). The Fatty Acid-Binding Protein, aP2, Coordinates Macrophage Cholesterol Trafficking and Inflammatory Activity. J. Biol. Chem. 280 (13), 12888–12895. 10.1074/jbc.m413788200 15684432 PMC3493120

[B13] Ren J. L. Chen Y. Zhang L. S. Zhang Y. R. Liu S. M. Yu Y. R. (2021). Intermedin 1-53 Attenuates Atherosclerotic Plaque Vulnerability by Inhibiting CHOP-Mediated Apoptosis and Inflammasome in Macrophages. Cel Death Dis. 12 (5), 1–6. 10.1038/s41419-021-03712-w PMC8088440 33934111

[B14] Ridker P. M. Genest J. Boekholdt S. M. Libby P. Gotto A. M. Nordestgaard B. G. (2010). HDL Cholesterol and Residual Risk of First Cardiovascular Events after Treatment with Potent Statin Therapy: an Analysis from the JUPITER Trial. Lancet 376, 333–339. 10.1016/S0140-6736(10)60713-1 20655105

[B15] Roger V. L. (2015). Cardiovascular Diseases in Populations: Secular Trends and Contemporary Challenges-Geoffrey Rose Lecture, European Society of Cardiology Meeting 2014. Eur. Heart J. 36 (32), 2142–2146. 10.1093/eurheartj/ehv220 25994744

[B16] Roh J. Chang C. L. Bhalla A. Klein C. Hsu S. Y. (2004). Intermedin Is a Calcitonin/calcitonin Gene-Related Peptide Family Peptide Acting through the Calcitonin Receptor-like Receptor/receptor Activity-Modifying Protein Receptor Complexes. J. Biol. Chem. 279 (8), 7264–7274. 10.1074/jbc.M305332200 14615490

[B17] Rolph M. S. Young T. R. Shum B. O. Gorgun C. Z. Schmitz-Peiffer C. Ramshaw I. A. (2006). Regulation of Dendritic Cell Function and T Cell Priming by the Fatty Acid-Binding Protein AP2. J. Immunol. 177 (11), 7794–7801. 10.4049/jimmunol.177.11.7794 17114450

[B18] Seidelmann S. B. Lighthouse J. K. Greif D. M. (2014). Development and Pathologies of the Arterial wall. Cell Mol Life Sci 71 (11), 1977–1999. 10.1007/s00018-013-1478-y 24071897 PMC11113178

[B19] Taggart M. (2010). Vascular Function in Health and Disease Review Series. J. Cel Mol Med 14 (5), 1017. 10.1111/j.1582-4934.2010.01050.x PMC3822735 20219014

[B20] Yudkin J. S. Kumari M. Humphries S. E. Mohamed-Ali V. (2000). Inflammation, Obesity, Stress and Coronary Heart Disease: Is Interleukin-6 the Link?: Is Interleukin-6 the Link. Atherosclerosis 148 (2), 209–214. 10.1016/s0021-9150(99)00463-3 10657556

[B21] Zhang S. Y. Xu M. J. Wang X. (2018). Adrenomedullin 2/intermedin: a Putative Drug Candidate for Treatment of Cardiometabolic Diseases. Br. J. Pharmacolapr 175 (8), 1230–1240. 10.1111/bph.13814 PMC5867024 28407200

[B22] Zhang X. Gu L. Chen X. Wang S. Deng X. Liu K. (2012). Intermedin Ameliorates Atherosclerosis in ApoE Null Mice by Modifying Lipid Profiles. Peptides 37 (2), 189–193. 10.1016/j.peptides.2012.07.011 22910189

