# Supplementary figures and images for: Intermedin Inhibits the Ox-LDL–Induced Inflammation in RAW264.7 Cells by Affecting Fatty Acid–Binding Protein 4 Through the PKA Pathway
Source: Front Pharmacol. 2021 Dec 1;12:724777. doi: 10.3389/fphar.2021.724777 (PMC8671820; doi:10.3389/fphar.2021.724777)

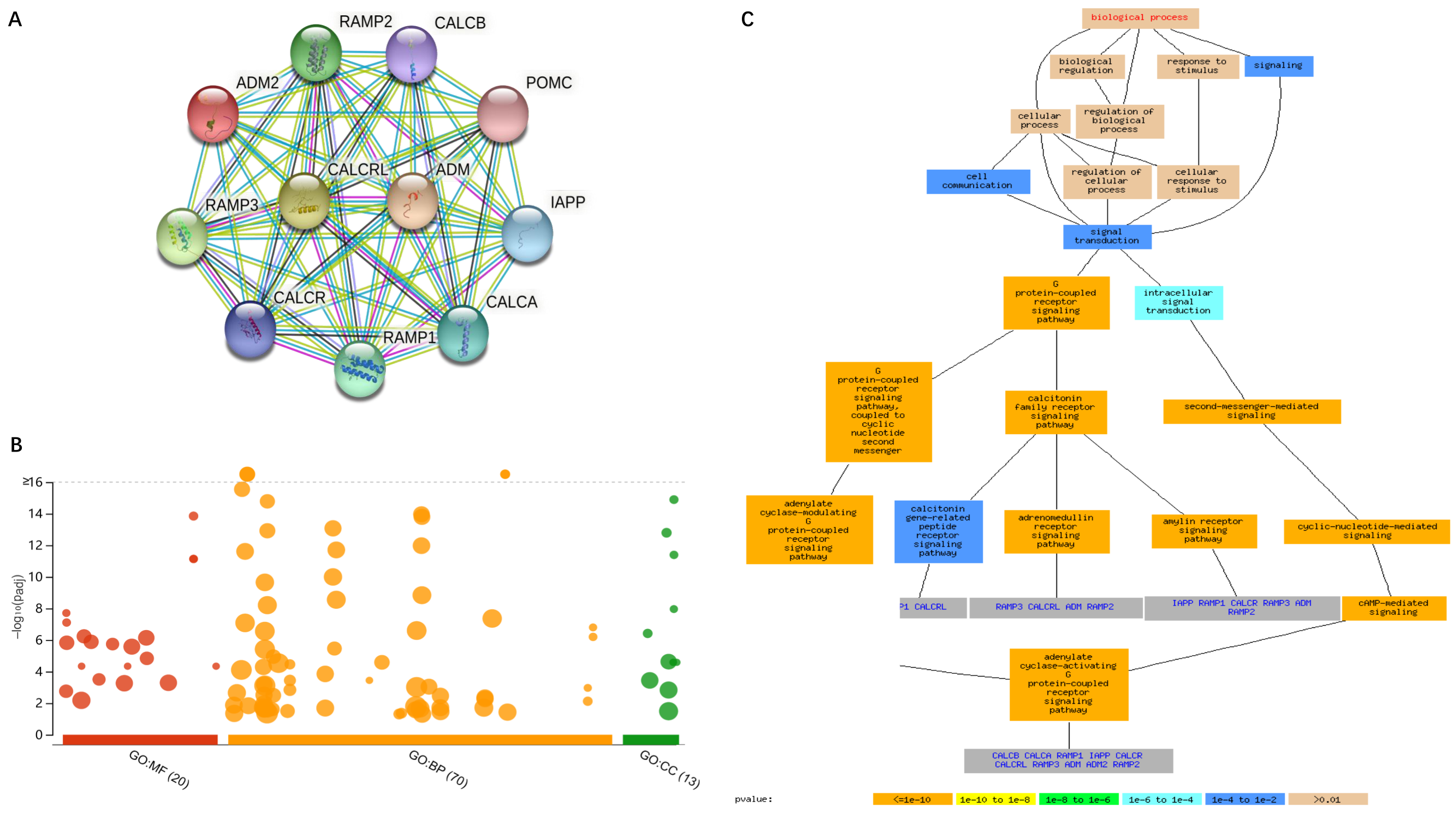

Supplement: Supplementary file 1 [file Image1.TIFF]

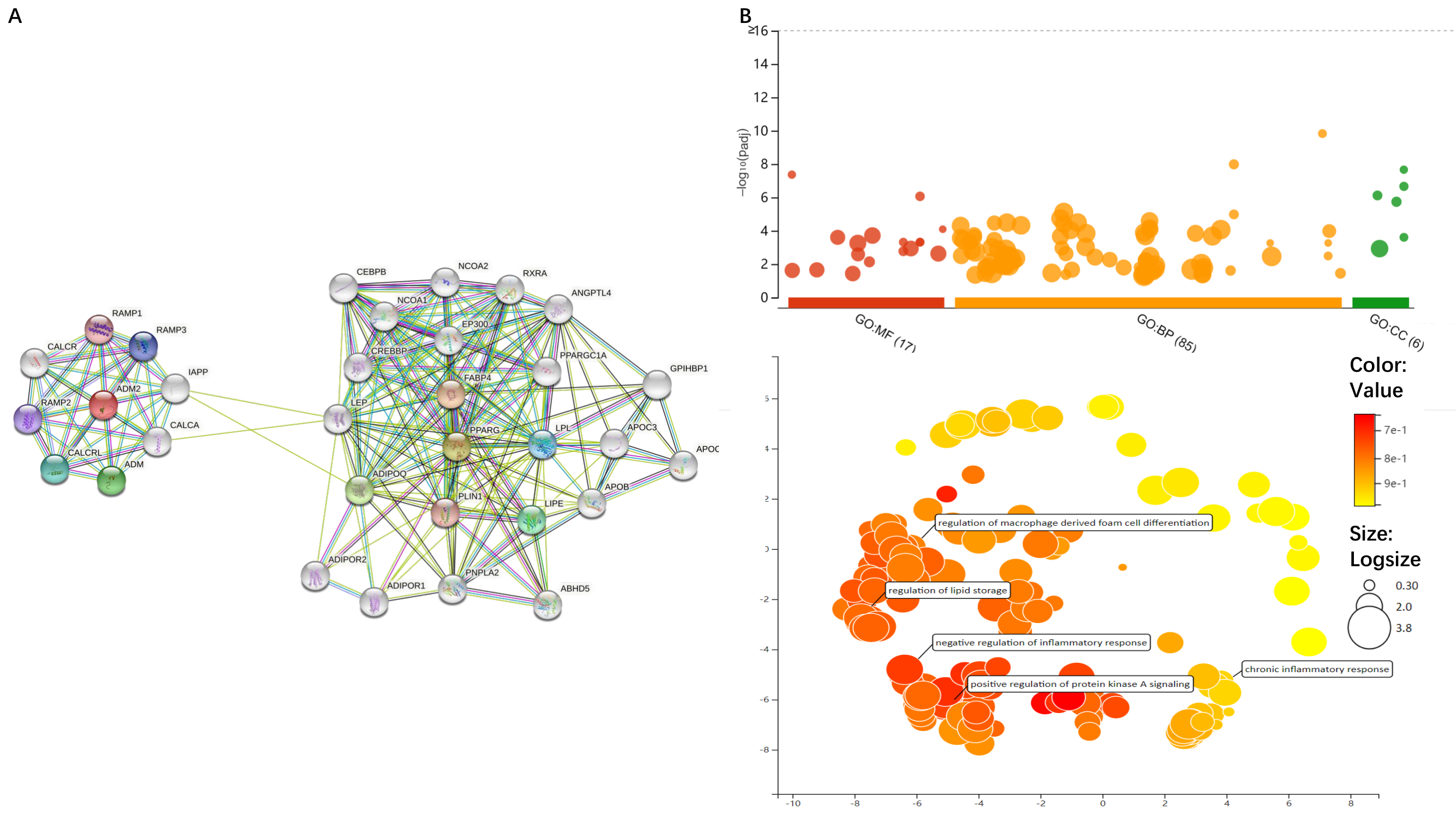

Supplement: Supplementary file 4 [file Image2.TIFF]
